# Efficacy and Safety of Oral TDF-Based Pre-exposure Prophylaxis for Men Who Have Sex With Men: A Systematic Review and Meta-Analysis

**DOI:** 10.3389/fphar.2018.00799

**Published:** 2018-09-04

**Authors:** Xiaojie Huang, Jianhua Hou, Aixin Song, Xinchao Liu, Xiaodong Yang, Junjie Xu, Jing Zhang, Qinghai Hu, Hui Chen, Yaokai Chen, Kathrine Meyers, Hao Wu

**Affiliations:** ^1^Center for Infectious Diseases, Beijing You'an Hospital, Capital Medical University, Beijing, China; ^2^Infectious Diseases Department, Peking Union Medical College Hospital, Beijing, China; ^3^Key Laboratory of AIDS Immunology of National Health and Family Planning Commission, Department of Laboratory Medicine, The First Affiliated Hospital, China Medical University, Shenyang, China; ^4^School of Biomedical Engineering, Capital Medical University, Beijing, China; ^5^Department of Infectious Diseases, Chonging Public Health Medical Center, Chongqing, China; ^6^The Aaron Diamond AIDS Research Center, New York, NY, United States

**Keywords:** HIV, pre-exposure prophylaxis, TDF, men who have sex with men, meta-analysis

## Abstract

**Background:** Pre-exposure prophylaxis (PrEP) is used as an HIV prevention method by people at substantial risk of HIV infection. This systematic review and meta-analysis evaluates current clinical evidence for use of oral TDF-based PrEP among men who have sex with men.

**Methods:** A comprehensive literature search in PubMed, web of science, Google Scholar and ClinicalTrials.gov was performed. A random-effects meta-analysis was conducted using the event rate (ER) for estimation of the incidence of HIV and grade 3 or 4 adverse events (AE) among PrEP arm and using risk ratio (RR) for comparison of incidence of HIV and grade 3 or 4 AE between PrEP recipients and PrEP non-users. Blood-based adherence levels were also divided into three categories with reference to previous meta-analysis. Subgroup meta-analysis was also performed to evaluate whether blood-based adherence levels moderated the effect of TDF-based PrEP on HIV incidence. Narrative review was used due to inconsistent measurements of risk behavior and drug resistance. This review is registered on the PROSPERO database (CRD42017077965).

**Results:** Fourteen studies were included in the review. Oral TDF-based PrEP significantly reduced HIV incidence with minimum drug resistance and tolerable safety risks (HIV incidence, ER = 1.1%, 95% CI 0.6–2.0%, *p* < 0.001, RR = 0.244, 95% CI 0.111–0.537, *p* < 0.001 and grade 3 or 4 AEs, ER = 13.0%, 95% CI 9.9–16.9%, *p* < 0.001, RR = 1.059, 95% CI 0.824–1.362, *p* = 0.653). Oral TDF-based PrEP was more effective in reducing HIV incidence with high levels of blood-based PrEP adherence (ER, 0.4%) compared to moderate adherence (2.9%; *p* < 0.001). Most studies found no association between PrEP use and self-reported sexual behavior.

**Conclusion:** Oral TDF-based PrEP is an effective intervention to prevent against HIV infection among MSM. Well-designed implementation science studies that integrate sociobehavioral and biomedical interventions are needed to identify optimal PrEP delivery models in different populations to translate biomedical efficacy into real-world efficacy.

## Introduction

Around 1.8 million people became newly infected with HIV in 2016 globally, according to the Joint United Nations Program on HIV/AIDS (UNAIDS, 2016), indicating that tremendous efforts are needed to slow down the AIDS epidemic. Globally there continue to be rising rates of HIV incidence among men who have sex with men (i.e., gay and bisexual men) (Prejean et al., 2011; Phillips et al., 2013; Reback and Fletcher, 2014; Newsum et al., 2017), with the highest HIV incidence rates observed among MSM in countries such as China, Kenya, Thailand, and among young and minority MSM in the US (Beyrer et al., 2016).

Although the promotion of regular HIV testing and encouragement of condom use will remain essential preventative strategies for HIV infection, additional approaches for people who are unwilling or unable to use condoms consistently are needed. In 2012, WHO first published a PrEP guidance, which recommended the use of tenofovir (TDF) and emtritabine (FTC) for people who are at substantial risk for HIV (such as serodiscordant couples and MSM). In recent years, daily use of PrEP by populations at high risk of HIV infection has been recommended in guidelines from United States, Europe, Australia and South Africa. Previous meta-analyses and systematic reviews have indicated that while TDF/FTC-based oral PrEP protects (Fonner et al., 2016; Desai et al., 2017), the efficacy of PrEP may vary across populations.

Thus we conducted this systematic review and meta-analysis to specify the efficacy and safety of TDF/FTC-based oral PrEP specifically among MSM.

## Methods

The review was registered in the in the International Prospective Register of Systematic Reviews (PROSPERO, https://www.crd.york.ac.uk/PROSPERO/): CRD42017077965. The work was reported in accordance with the Preferred Reporting Items for Systematic Reviews and Meta-Analysis (PRISMA) (Moher et al., 2009). Detailed information of PRISMA checklist is included in Supplementary Table 1.

### Search strategy

A comprehensive literature search in PubMed, web of science, Google Scholar and ClinicalTrials.gov was performed. Search terms were intersections of PrEP related terms (pre-exposure prophylaxis OR preexposure prophylaxis OR antiretroviral prophylaxis OR preexposure chemoprophylaxis OR chemoprevention OR PrEP OR Truvada), disease terms (HIV OR AIDS) and target population terms (MSM OR gay OR men have sex with men). Reference lists of selected articles and related review articles were further screened. Additional searches in Google Scholar and ClinicalTrials.gov were also performed. The search was limited to English-written journal articles and conference abstracts. The full search strategy is shown in Supplementary Table 2.

### Selection criteria

**Figure 1:**
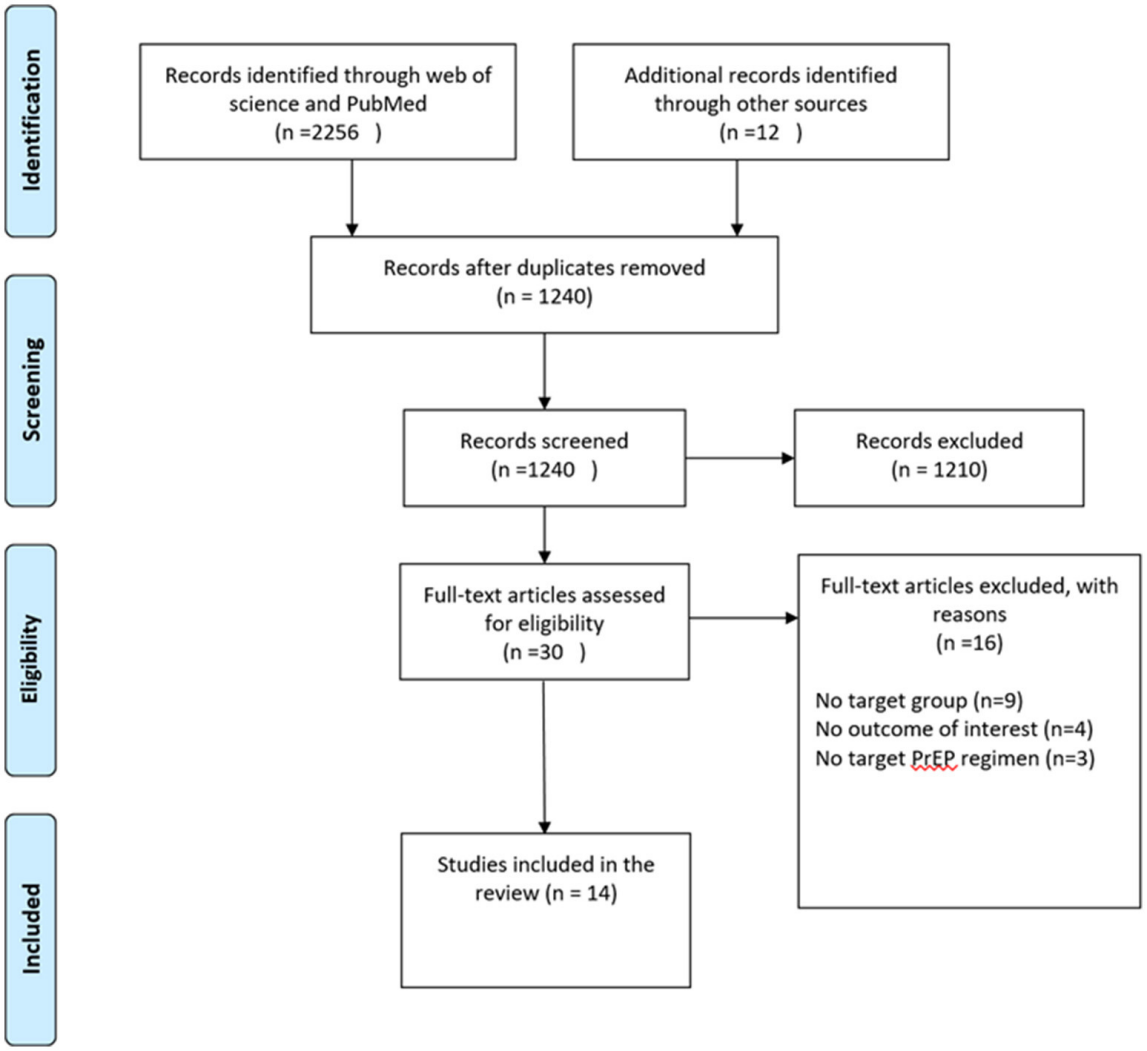
Flow diagram of this systematic review and meta-analysis.

To be included in the meta-analysis, the study had to (1) focus on MSM and transgender women; (2) evaluate the efficacy or safety of TDF-based PrEP; (3) provide sufficient data on at least one outcome of interest. Outcomes of interest included: HIV infection, grade 3 or 4 AEs and behavior (condom use, STI and number of sexual partners) and drug resistance. Studies were excluded if they were (1) case reports; (2) review articles or theoretical articles; (3) thesis, dissertation or book chapters. Two reviewers (JHH and AXS) initially selected search results based on titles and abstracts. The remaining articles were further selected by full-text assessment by JHH and AXS. Disagreements between reviewers about eligibility were resolved by discussion with XJH. The procedure of study selection and numbers of included and excluded studies is shown in Figure 1.

### Data extraction and code

Coding of outcome measures was done by two reviewers (JHH and AXS) based on previous meta-analysis reviews. Outcomes of interest included: HIV infection, grade 3 or 4 AEs, behavior (condom use, STI and number of sexual partners) and drug resistance mutation. Other information was also extracted from articles including article author, year of publication, study location, age of participants, sample size, study design, PrEP regimen, PrEP dose, and adherence. The results of quality assessment are also shown in Table 1.

**Table 1:** Study characteristic.

**Study name**	**Year of publication**	**Number of participants**	**Study Design**	**PrEP regimen**	**Dose**	**Comparison**	**Adherence**	**Location**
CDC Safety Study (Grohskopf et al., 2013)	2013	400	RCT	TDF	Daily	PrEP vs. placebo	94%	United States
NCT01632995 (Liu et al., 2016)	2016	557	Demonstration	FTC/TDF	Daily	PrEP group	80–86% from wk 4 to 48	San Francisco, California, and Miami, Florida and Washington DC United States
HPTN-069 (Gulick et al., 2017)	2016	406	RCT	FTC/TDF	Daily	Four PrEP arms (TDF/FTC arm used in the analysis)	83%,74% at wk 24,48	United States
Ipergay (Molina et al., 2015)	2015	400	Real world RCT	FTC/TDF	On demand	PrEP vs. placebo	Not reported	France and Canada
Ipergay OLE (Molina et al., 2017)	2017	361	Real world Cohort	FTC/TDF	On demand	Only PrEP group	Not reported	France and Canada
iPrEx (Grant et al., 2010)	2010	2499	RCT	FTC/TDF	Daily	PrEP vs. placebo	51%	Peru, Ecuador, South Africa, Brazil, Thailand, and United States
iPrEx OLE (Grant et al., 2014)^@^	2014	1603	Cohort	FTC/TDF	Daily	PrEP vs. no PrEP	71%	Peru, Ecuador, South Africa, Brazil, Thailand, and United States
KPSF (Volk et al., 2015)	2015	657	Real world Cohort	FTC/TDF	Daily	Only PrEP group	Not Reported	San Francisco, United States
PATH-PrEP (Landovitz et al., 2017)	2017	297	Cohort	FTC/TDF	Daily	PrEP and PEP cohorts: only PrEP cohort was analyzed	65.5%-83.4% from wk 4 to 48	Los Angeles, California
ATN082 Project PrEPare (Hosek et al., 2013)	2013	58	RCT	FTC/TDF	Daily	PrEP vs. placebo vs. no Pill	63.2% (wk 4) to 20% (wk 24)	United States
PROUD-pilot phase (I) (McCormack et al., 2016)	2016	545	Real world RCT	FTC/TDF	Daily	Immediate PrEP to delayed PrEP	Not reported	England
PROUD-Second phase (II)^$^	2017	255	Real world Second phase of PROUD	FTC/TDF	Daily	Delayed with PrEP (second phase) vs. Delayed without PrEP (first phase)	Not reported	England
ATN110 YSMS (Hosek et al., 2017)^&^	2017	200	Demonstration	FTC/TDF	Daily	PrEP group	Not reported	United States
HPTN073BSMS (Wheeler et al., 2016)	2016	226	Demonstration	FTC/TDF	Daily	PrEP vs. No PrEP	Not reported	Washington DC, Los Angeles and Chapel Hill, NC United States

*BSMS, black men who have sex with men; CDC, Center for Disease Control and Prevention; FTC, emtrictabine; KPSF, Kaiser Permanent Medical Center; OLE, open label extension; NA, not available, RAI, receptive anal intercourse; RG, reduced-glycerin; wk, week; YSMS, young men who have sex with men*.

$ *For PROUD-second phase: the deferred PrEP group in the second phase (PrEP use) was self-compared to the first phase (No PrEP).(with reference to 9th International AIDS Society Conference on HIV Science)*.

& *In this study, DBS results were translated into dosing categories previously used in PrEP trials with adult MSM. The proportion of daily use of PrEP ranges from 4.6 to 21.4% during 48 weeks follow-up. But in our meta-analysis, we do not use this translated data*.

@ *In this study, most participants were recruited from iPrEx (68 participants in ATN082, 2499 participants in iPrEx and 279 participants from US safety study;1603 participants in this cohort meet the criteria for PrEP), so we use iPrEx OLE to name this cohort study*.

### Data analysis

We adopted Comprehensive Meta-Analysis (CMA) Version 2.0 (Biostat, Englewood, New Jersey) to conduct a quantitative analysis. First, combined event rate (ER) was calculated using number of events and sample size of PrEP arms. A random-effects meta-analysis was conducted using the event rate (ER) for estimation of the incidence of HIV and grade 3 or 4 AEs among oral TDF-based PrEP arm and using risk ratio (RR) for comparison of incidence of HIV and grade 3 or 4 AEs between oral TDF-based PrEP users and non-users. For open label extensions (OLEs) of previous RCTs, narrative review was used in order to avoid calculating the results based on the same sample. The variation in effect sizes across studies was assessed by the homogeneity statistics, Q. *I*^*2*^ statistic was also used to estimate the observed proportion of the heterogeneity in observed variance (Higgins and Thompson, 2002). Begg rank correlation test was adopted to assess publication bias across studies when more than three comparisons were in the analysis (Begg and Mazumdar, 1994). In some cases, narrative review was also used due to inconsistent measurements of some outcomes of interest including condom use, STI and drug resistance. Blood-based adherence levels were also divided into three categories with high adherence, >70%; moderate, 40–70%; and low, <40% according to arithmetic average of each time point with reference to previous meta-analysis (Fonner et al., 2016). Subgroup meta-analysis was performed to assess the difference among different blood-based adherence levels on HIV incidence.

### Role of the funding resource

The funder of the study had no role in study design, data collection, data analysis, data interpretation, or writing of the report. The corresponding authors had full access to all the data in the study and had final responsibility for the decision to submit for publication.

## Results

### Characteristic of included studies

Overall we identified 14 eligible studies in this review, whose sample size ranged from 58 to 2,499. We included six RCTs and eight OLEs or demonstration projects. All participants were adults over 18. The most commonly-used PrEP regimen was TDF in combination with FTC. Seven studies reported detectable blood-based drug component with a range from 51 to 94% of all populations. Thirteen studies examined daily oral PrEP, but Ipergay and its OLE examined non-daily dosing strategy (on demand: two pills taken 2–24 h before sex act and one pill taken 24 h after sexual intercourse and one pill taken 48 h after sex act). These studies reported a variety of outcomes including HIV incidence, grade 3 or 4 AEs, self-reported risk behavior and number of sex partners, STI and drug resistance. Detailed information for included studies is shown in Table 1.

### HIV incidence

**Figure 2:**
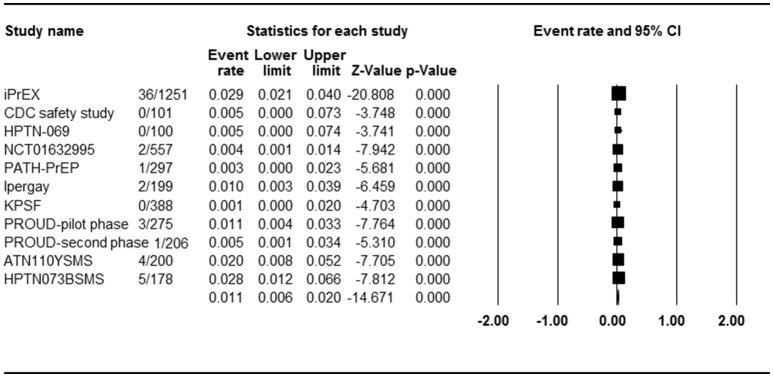
Forest plot for overall analysis of PrEP and HIV incidence (ER).

Eleven studies with 11 PrEP arms reported the effect of PrEP on HIV incidence, so ER of each study could be analyzed. Begg rank correlation test showed no significant publication bias (Kendall's tau = −0.309, *p* = 0.09). As shown in Figure 2, the combined ER of HIV incidence in PrEP arm was 1.1% (95% CI 0.6–2.0%, *p* < 0.001). Results revealed significant heterogeneity across studies [*Q*_(10)_ = 24,524, *p* = 0.006, *I*^2^ = 59.2%].

**Figure 3:**
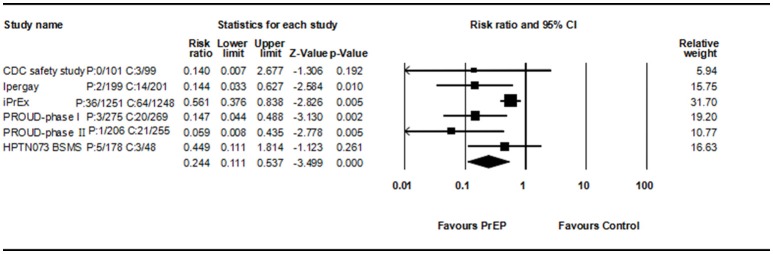
Forest plot for overall analysis of PrEP and HIV incidence (RR).

Among eleven studies, six studies compare PrEP with no PrEP or placebo, so RR of each study could be analyzed. All consistently reported a decreased HIV incidence rate. Begg rank correlation test showed no significant publication bias (Kendall's tau = −0.33, *p* > 0.05). The combined RR of PrEP on HIV incidence was 0.244 (95% CI 0.111–0.537, *p* < 0.001, Figure 3). Results revealed significant heterogeneity across studies [*Q*_(5)_ = 11.236, *p* = 0.047, *I*^2^ = 55.5%].

Regarding open label extensions, results from iPrEx US-based OLE cohorts showed that PrEP use was associated with reductions in HIV incidence when comparing PrEP recipients (1.8 infections per 100 person, 95% CI 1.5–4.5) to PrEP non-recipients (2.6 infections per 100 persons, 95% CI 1.3–2.6). Results from Ipergay OLE showed that the overall HIV incidence in this follow-up phase was 0.19 per 100 person-years of follow-up (95% CI 0.01–1.08).

When stratified by blood-based adherence, there were four studies classified as high-adherence group, one study as moderate-adherence group and no studies as low-adherence group. Adherence significantly moderated the efficacy of PrEP [*Q*_(1)_ = 14.939, *p* < 0.001]. The ER for HIV incidence for high adherence was 0.4% (95% CI 0.1–1.0%) and 2.9% (95% CI 2.1–4%) for moderate adherence.

Detailed information of HIV infection is also shown in Table 2.

**Table 2:** Effects of PrEP on HIV infection.

**Study**	**Outcome**		**Risk reduction^#^**
	**PrEP arm (TDF-based regimen)**	**Control**	
CDC safety study	0 infections among 101 persons (PrEP immediate arm)	3 infection among 99 persons (placebo immediate arm)	
NCT01632995	2 infections among 557 persons during follow-up		
HPTN-069	0 infections among 100 persons (PrEP immediate arm)		
Ipergay	2 infection among 199 persons	14 infection among 201 persons	86% in relative reduction (95% CI 40–98, *p* = 0.002)
Ipergay OLE	0.19 per 100 person-year	6.6 per 100 person-years	97% in relative reduction (95% CI 81–100)
iPrEx	36 infections among 1251 persons	64 infection among 1225 persons	44% in relative reduction (95% CI 15–63, *p* = 0.005)
iPrEx OLE	1.8 infections per 100 person-year	2.6 infection per 100 person-year	
KPSF	0 infections among 657 initiating PrEP		
PATH-PrEP	1 infection among 297 persons		
PROUD-pilot phase	3 infections among 275 persons	20 infections among 269 persons	86% in relative reduction (95%CI 64–96, *p* = 0.001)
PROUD-second phase	1 infection among 206 persons	20 infections among 269 persons	
ATN110 YSMS	4 infections among 200 persons		
HPTN073BSMS	5 infections among 178 persons		

# *We reported risk reduction based on original papers*.

### Grade 3 or 4 adverse events

**Figure 4:**
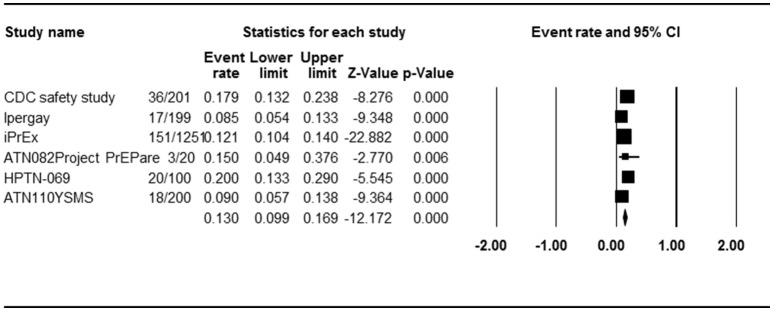
Forest plot for overall analysis of PrEP and any AEs (ER).

Six studies with six PrEP arms reported the effect of PrEP on grade 3 or 4 AE, allowing us to calculate the ER of each study. Begg rank correlation test showed no significant publication bias (Kendall's tau = −0.2, *p* = 0.286). As shown in Figure 4, the combined ER of grade 3 or 4 AEs in PrEP arm was 13.0% (ER, 95% CI 9.9–16.9%, *p* < 0.001). Results revealed significant heterogeneity across studies [*Q*_(5)_ = 15,579, *p* = 0.008, *I*^2^ = 67.9%].

**Figure 5:**
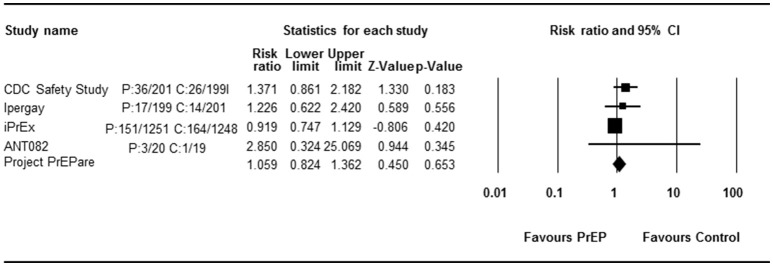
Forest plot for overall analysis of PrEP and grade 3 or 4 AEs (RR).

Four RCT studies reported the effect of PrEP on grade 3 or 4 AEs. All consistently reported a comparable AE rate in the PrEP and control arms. Begg rank correlation test showed no significant publication bias (Kendall's tau = 0.333, *p* = 0.497). The combined effect size of PrEP on grade 3 or 4 AEs was 1.059 (RR, 95% CI 0.824–1.362, *p* = 0.653, Figure 5). Results revealed no significant heterogeneity across studies [*Q*_(3)_ = 3.654, *p* = 0.301, *I*^2^ = 17.9%].

Regarding open label extensions, results from Ipergay OLE showed that the grade 3 or 4 AE rate in this follow-up phase was 11% (40/361; 40 of the total participants have grade 3 or 4 AEs during OLE). The iPrEx OLE cohorts did not provide specific data on grade 3 or 4 AEs.

Summary of grade 3 or 4 AEs in each study is also shown in Supplementary Table 3.

### Behavior change

Condom use, STI, and number of sex partners were summarized in this review as indices of behavior change. Quantitative synthesis was improper due to inconsistent measurements of condom use, STI and number of sex partners in these studies. Thus narrative description of each outcomes is included in this review.

Condom use was reported in five RCTs (iPrEx, Ipergay, CDC safety study, PROUD, and Project PrEPare), and six longitudinal observational studies (iPrEx OLE, Ipergay OLE, PATH-PrEP, KPSF, ANT110YSMS, and NCT01632995). Among RCT studies comparing PrEP and no-PrEP, results from these studies showed similar change between different arms and for observational studies, condom use was stable or slightly decreased in PrEP arms (Grant et al., 2010, 2014; Hosek et al., 2013; Liu et al., 2013; Molina et al., 2015; McCormack et al., 2016). One observational study (Ipergay OLE) reported a significant rise in condomless anal sex at the last sexual intercourse.

Incidence of STIs was recorded in seven studies (iPrEx OLE, Ipergay OLE, KPSF, NCT01632995, PATH-PrEP, PROUD-pilot phase, ATN110YSMS). Inconsistent results were reported across studies. Two studies reported comparable rates of STIs between PrEP users and non-users (iPrEx OLE and PROUD-pilot phase). Three studies reported stable or decreased STI incidence rates during follow-up (Ipergay OLE, NCT01632995, and ATN110YSMS). One study demonstrated a parabolic distribution over the follow-up phase (PATH-PrEP). One study showed a slightly increase trend during follow-up (KPSF).

Number of sex partners was reported in four RCTs (iPrEx, Ipergay, CDC safety study, and PROUD) and four longitudinal observational studies (iPrEx OLE, Ipergay OLE, NCT01632995, and KPSF). Number of sex partners was stable or slightly decreased over time in the PrEP arms and similar between PrEP users and PrEP non-users despite different study designs and measurements of outcome (Grant et al., 2010, 2014; Liu et al., 2013; Molina et al., 2015; McCormack et al., 2016).

Detailed information of condom use, STI and number of sexual partners is recorded in Tables 3–5 respectively.

**Table 3:** Effects of PrEP on condom use.

**Study name**	**Outcome**	**Description**
**RCTS: PrEP vs. NO PrEP**		
CDC safety study	CAS CASPU	CAS: no statistical difference between immediate vs. delayed arms during months 3–9 (*p* = 0.10) and no significant change when delayed group initiated study drug 0.42) CASPU: remained stable or decreased [2.02 at baseline vs. 1.51 during months 3–9 (*p* = 0.22) and 2.02 at baseline vs. 1.37 during months 12–24 (*p* = 0.05)]
iPrEx study	Percent of receptive anal partners with which condoms used	No significant treatment by visit interaction (*p* = 0.36) with 50.38% in TDF-FTC and 51.04% in placebo at baseline plus 73.98% in TDF-FTC and 83.64% in placebo at follow-up
Project PrEPare	CAS	No significant differences among the 3 treatment groups across visits; Insignificant trend from baseline to week 24 of decreasing condomless anal sex across all treatment arms.
Ipergay	CAS	No significant differences in the total number of episodes of sexual intercourse in the 4 weeks before visits (*p* = 0.07), in the proportion of episodes of CRAS (*p* = 0.40), or in the proportion of episodes of CAS during the most recent sexual intercourse (*p* = 0.90) across groups.
**OBSERVATIONAL STUDIES: CHANGE DURING PrEP USE or COMPARISON BETWEEN PRE/POST PrEP**		
iPrEx OLE	CAS	Decreased from 34% (377/1115) to 25% (232/926) among PrEP recipients (*p* = 0.006), and from 27 (101/369) to 20% (61/304) among non-recipients (*p* = 0.03). Decreases in CRAS and CIAS were similar across groups (*p* = 0.95 and *p* = 0.56).
Ipergay OLE	CAS at the last sexual intercourse	CAS: significant increase from 77%(136/176) at baseline to 86%(66/76) at 18 months' follow-up (*p* = 0.004)
KPSF	Condom use	Condom use: unchange in 56% of PrEP users, decrease in 41% of PrEP users, and increase in 3% of PrEP users
NCT01632995	CAS	CAS:baseline:165/557(65.5%);remained stable (65.6%) during follow-up (*p* = 0.99)
PATH-PrEP	CAS	CAS: decreased over the first 24 weeks of study, and then were stable thereafter

*CAS, condomless anal sex; CASPU, condomless anal sex with HIV positive/unknown status partner; CRAS, condomless receptive anal intercourse; CIAS, condomless insertive anal intercourse; PY, person-years*.

**Table 4:** Effects of PrEP on STIs.

**Study name**	**Outcome**	**Description**
**RCTS: PrEP vs. No PrEP**		
PROUD-pilot phase	STI	Proportion with confirmed rectal chlamydia/gonorrhea was similar in immediate (29%) and delayed (27%) (*p* = 0.50) arms.
**OBSERVATIONAL STUDIES: CHANGE DURING PrEP USE or COMPARISON BETWEEN PRE/POST PrEP**		
iPrEx OLE	Syphilis incidence	Syphilis incidence was similar among PrEP users and non-users (HR 1.35, 95 CI 0.83–2.19). PrEP no-user:7.2 infections per 100 PY, PrEP no-user:5.4 infections per 100 PY
Ipergay OLE	Incidence of first bacterial STIs	Remain stable from 49.1 per 100 PY (95% CI 41.6–57.6) at RCT phase to 59.0 per 100 PY0 (95% CI 50.1–69.0) at open phase (*p* = 0.11)
KPSF	Multiple STIs	STIs: 187/657 PrEP initiators: at least 1 STI during follow-up; 78/657 PrEP initiators: multiple STIs (range 2–10); after 6 months of PrEP use, 30% of PrEP users with any STI (95% CI 26–35%), after 12 months of PrEP use, 50% of PrEP users with any STI (95% CI 43–56%)
NCT01632995	Multiple STIs	Overall STI incidence:90 per 100 PY (81–99); incidence remain stable across quarterly (*p* > 0.1)
PATH-PrEP	STI	STI:177(46.4%) participants with 175 STIs over 48 weeks follow-up, with a parabolic distribution over the follow-up phase (Baseline = 20.3%, 24 weeks = 17.7%,48 weeks = 28%)
ATN110YSMS	STI	Decreased from 76.48/100 PY at first 24 weeks to 60.99/100 PY in the latter 24 weeks

*STI, sexual transmitted infection, PY, person years*.

**Table 5:** Effects of PrEP on number of sexual partners.

**Study name**	**Outcome**	**Description**
**RCTS: PrEP vs. NO PrEP**		
CDC safety study	Mean number of male sex partners in past 3 months	Decreased significantly from 7.25 at baseline to 6.02 during months 3–9 and 5.71 during months 12–24 (*P* < 0.001). Declines were similar between the immediate vs. delayed arms during months 3–9 (*P* value for interaction = 0.67) Mean number did not differ in months 12–24 vs. months 3–9 with initiation of study drug in delayed arm (*p* = 0.22) or drug continuation in immediate arm (IRR = 0.96, *P* = 0.56). Mean number of positive or unknown HIV-status partners declined from 4.17 at baseline to 3.51 during months 3–9 (*P* = 0.04) and 3.37 during months 12–24 (*P* = 0.01).
iPrEx study	Mean number of receptive anal partners	Baseline: FTC-TDF: 12.21 (SE = 0.81); Placebo: 11.21 (SE = 0.81) Follow-up (132 weeks): FTC-TDF: 3.47 (SE = 0.81); Placebo: 5.71 (SE = 1.59) Wald test of the treatment by visit interaction: *p* = 0.97
Ipergay	Number of sexual partners in the past 2 months	There was a slight but significant decrease in the number of sexual partners within the past 2 months in the placebo group as compared with the TDF-FTC group
**OBSERVATIONAL STUDIES: CHANGE DURING PrEP USE OR COMPARISON BETWEEN PRE/POST PrEP**		
iPrEx OLE	Number of sexual partners	Numbers of sexual partners were much the same in the each groups (*p* = 0.64)
Ipergay	Number of sexual partners in the past 2 months	There was a slight but significant decrease in the number of sexual partners within the past 2 months in the placebo group as compared with the TDF-FTC grou
Ipergay OLE	Number of sexual partners during the 2 months before visit Total number of sexual intercourses in the 4 weeks before visits	No significant change during the 2 months before visits (*p* = 0.42)and the total number of sexual intercourses in the 4 weeks before visits (*p* = 0.12)
KPSF	Number of sexual partners every 1–3 months after PrEP initiation	Unchange:74% of PrEP users, decrease:15% of PrEP users, and increase:11% of PrEP users after 6 months
NCT01632995	Number of anal sex partners in the past three months	Declined from 10.9 at baseline to 9.3 at week 48 (*p* = 0.04)
PROUD-pilot phase	Number of anal sex partners in last 90 day	At baseline: immediate arm: 10.5 (5–20); delayed arm: 10 (4–20) and at month 12: immediate arm: 10 (3–24); delayed arm: 8 (3–15)

### Drug resistance

Drug resistance was reported in six studies (CDC safety study, iPrEx study, iPrEx OLE, PROUD-pilot phase, NCT01632995, and ATN110YSMS). No drug resistance was found to be associated with TDF at enrollment or during the trial or follow-up phase. FTC-related mutation was observed at enrollment or during the trial phase in four studies with minimum event rate. Specifically, FTC-related drug resistance was found at enrollment in one studies (iPrEx study) and after enrollment in two studies (iPrEx OLE and NCT01632995). For PROUD-Pilot phase study, FTC- related mutation was found in two of the three participants who initiated TDF/FTC PrEP with a reactive test at enrolment or at the 4-week visit. Detailed information is recorded in Table 6.

**Table 6:** Effects of PrEP on drug resistance.

**Study name**	**PrEP regimen**	**Drug resistance**		**Event rate**
		**TDF-related**	**FTC-related**	
ATN110YSMS	TDF/FTC	No	No	0
CDC safety study	TDF	No	No	0
iPrEx study	TDF/FTC	No	Enrollment phase: *n* = 3 (2 in PrEP group and one in placebo group) Trial phase: No	0.12%
iPrEx OLE	TDF/FTC	No	Met184Val (*n* = 1)	0.06%
NCT01632995	TDF/FTC	No	M184MI (*n* = 1)	0.17%
PROUD-pilot phase	TDF/FTC	No	At enrolment or the 4-week visit (*n* = 2): Met184Ile/Met, Met184Ile/Val/Met	0.36%

## Discussion

This systematic review provides synthesized estimates of HIV incidence, grade 3 or 4 AEs, self-reported risk behavior, STI and drug resistance among MSM using TDF-based PrEP. The overall estimates are useful for targeted prevention efforts among key population.

Our systematic review shows that PrEP significantly reduced HIV incidence, with low rates of HIV incidence among PrEP users, indicating the utility of this biomedical HIV prevention intervention in curbing HIV transmission among MSM. The findings in this meta-analysis are consistent with a previous review that evaluated the efficacy of PrEP across all populations (Fonner et al., 2016). As would be expected, PrEP was most effective in reducing HIV incidence among users with high levels of PrEP adherence. Adherence-support interventions for PrEP should be designed, building on the experience of HIV treatment studies that adopted multiple behavioral interventions to help improve and maintain treatment adherence (Popeleches et al., 2011; Ingersoll et al., 2015). Psychological counseling which focus on the development of coping strategies (i.e., Life Steps counseling in Partners PrEP Ancillary Adherence Study and Project PrEPARE and Next Step counseling in iPrEx OLE) should also be tested as strategies to increase adherence to PrEP medication (R Amico et al., 2012; Psaros et al., 2014; Taylor et al., 2017). Moreover, studies have demonstrated that some MSMprefer long-acting injectable formulation, which might also be beneficial for improving PrEP adherence (Meyers et al., 2017). Thus future studies are needed to evaluate the efficacy of PrEP in combination with suitable behavioral and psychological intervention or different PrEP formulations among MSM.

Regarding grade 3 or 4 AEs, no significant difference was detected between PrEP users and placebo/PrEP non-users. Common AEs of TDF-based regimen were nausea, diarrhea, back pain, headache, creatinine elevation, depression, potential bone and renal toxicity (Grant et al., 2010; Liu et al., 2011; Grohskopf et al., 2013; McCormack et al., 2016). Future PrEP studies should monitor these side effects and clinicians should treat these side effects immediately, which is likely to promote adherence to PrEP, both during future trials and in clinical practice.

With respect to changes in self-reported risk behavior associated to PrEP use, no significant difference was detected between PrEP users and placebo/PrEP non-users. In our meta-analysis, PrEP did not cause risk compensation in self-reported sex acts, either in the form of decreased condom use or increased number of sex partners. These results are consistent with a recent systematic review which includes both qualitative and quantitative survey studies (Freeborn and Portillo, 2017). But these results should be treated with caution, because self-report is often influenced by social desirability, which means individuals tend to report fewer risk behaviors in order to maintain a good social impression (Zenilman et al., 1995; Difranceisco et al., 1998). Mixed methods including the use of social desirability scales, the rating of item desirability, the use of forced-choice items and the randomized response technique to evaluate and decrease social desirability should be used in future studies (Nederhof, 1985). Selection bias might be a reason for the heterogeneity of STI incidence across studies (Jenness et al., 2017). For example, some studies reported PrEP users may be at higher risk for STIs prior to PrEP initiation and continue high risk behavior during PrEP use (Montano et al., 2017). In fact, the reduction of HIV incidence in the presence of stable or increased STI incidence suggests that these TDF-based PrEP studies in fact enrolled exactly the right population to benefit from biomedical HIV prevention. The relationship between PrEP and STI infection is unclear and further studies are needed to determine the potential effects of selection bias and risk compensation.

Regarding drug resistance, no drug resistance was found to be associated with TDF at enrollment or during the trial, supporting the evidence that TDF is a safe component in PrEP. In our review, we found FTC-related drug resistance might be more easily detected at enrollment and during the trial. Two special cases should not be ignored: one case was infected with multidrug resistance despite long-term use of PrEP and another acquired wild-type HIV virus despite consistent detectable drug levels (Knox et al., 2016; Elske and Godelieve, 2017). Overall drug resistance has rarely occurred among PrEP users, which indicates oral TDF in combination with FTC offers safe prevention for key populations. Moreover drug resistance due to daily PrEP might be associated with adherence. While such real world data does not exist, in one study using mathematic modeling, results have suggested 17–23% infected participants could virologically fail treatment as a result of past PrEP use or transmitted resistance to PrEP with moderate adherence (Dimitrov et al., 2016). Whether real world experience will bear out this model remains to be seen. Nevertheless, effective methods to support adherence should be provided and adherence should be monitored by researchers during PrEP demonstration studies in order to both reduce HIV infection and drug resistance. A urine-based drug detection assay currently under development as a point of care test could be useful tool to monitor PrEP adherence and identify PrEP users who require additional adherence support (Koenig et al., 2017). While a baseline HIV RNA test to rule out acute infection should be included in a clinical trial in order to decrease the risk of secondary resistance, the high cost of HIV RNA test may make the adoption of HIV RNA test before initiation of PrEP difficult to achieve in real world clinical practice. New testing algorithms may be needed in the PrEP era to minimize PrEP initiation during acute infection and to correctly diagnosis HIV in the context of PrEP-use.

Several limitations in the analysis should also be addressed. First, the limited number of studies included for some results could limit the ability to generalize the results to other settings. Future studies should thus adopt comprehensive evaluations including HIV infection, AE monitoring, risk behavior and drug resistance. Secondly, measurement for some outcomes (i.e., risk behavior) is inconsistent across studies, making it difficult to quantitatively analyze the effect of PrEP on risk compensation and drug resistance. Agreement to and use of a common set of behavioral measures would greatly enhance the field's ability to make meaningful comparisons across studies.

In summary, the results of our global meta-analysis suggest that oral TDF-based PrEP is effective at reducing HIV infection among MSM at substantial risk of HIV infection. Moreover, self-reported behavioral measurements indicate no significant risk compensation occurred in PrEP group. According to our results, clinicians should carefully monitor PrEP adherence and high risk sexual behaviors by self-reported questionnaires and blood-based laboratory tests. In resource limited countries, it might be an efficient and useful social health policy to integrate PrEP into medical insurance. Minimum drug resistance is associated with PrEP use. But results should be cautiously interpreted because of limited comparisons for some outcomes. Well-designed studies with large sample size are needed to optimize oral PrEP delivery and plan for other PrEP formulations. Consistent and reliable measurements of key outcomes (such as condom use and number of sexual partners) should also be used in the future studies.

## Author contributions

HW, XH, and JH conceptualized the study, and developed the research protocol. JH and AS identified articles for full-test review and extracted data that matched inclusion criteria. JH did the statistical analyses with input from XL and XY. All authors contributed to the writing of the manuscript. KM, XH, and JH polished and revised the manuscript.

### Conflict of interest statement

The authors declare that the research was conducted in the absence of any commercial or financial relationships that could be construed as a potential conflict of interest.
