# Supplementary material for: Efficacy and Safety of Oral TDF-Based Pre-exposure Prophylaxis for Men Who Have Sex With Men: A Systematic Review and Meta-Analysis
Source: Front Pharmacol. 2018 Sep 4;9:799. doi: 10.3389/fphar.2018.00799 (PMC6131617; doi:10.3389/fphar.2018.00799)
Supplement: Supplementary file 1 [file Table_1.DOCX]

**Contents**

[**Table 1 PRISMA checklist** 2](#_Toc505210167)

[**Table 2 Full Search Strategy** 6](#_Toc505210168)

[**Table 3 Summary of Grade 3 or 4 adverse event** 7](#_Toc505210169)

# **Table 1 PRISMA checklist**

| **Section/topic** | **#** | **Checklist item** | **Reported on page #** |
| --- | --- | --- | --- |
| **TITLE** | | |  |
| Title | 1 | Identify the report as a systematic review, meta-analysis, or both. | Page 1 Title Page |
| **ABSTRACT** | | |  |
| Structured summary | 2 | Provide a structured summary including, as applicable: background; objectives; data sources; study eligibility criteria, participants, and interventions; study appraisal and synthesis methods; results; limitations; conclusions and implications of key findings; systematic review registration number. | Page 2 Abstract Page |
| **INTRODUCTION** | | |  |
| Rationale | 3 | Describe the rationale for the review in the context of what is already known. | Page 6 |
| Objectives | 4 | Provide an explicit statement of questions being addressed with reference to participants, interventions, comparisons, outcomes, and study design (PICOS). | Page 6 |
| **METHODS** | | |  |
| Protocol and registration | 5 | Indicate if a review protocol exists, if and where it can be accessed (e.g., Web address), and, if available, provide registration information including registration number. | Page 6 |
| Eligibility criteria | 6 | Specify study characteristics (e.g., PICOS, length of follow-up) and report characteristics (e.g., years considered, language, publication status) used as criteria for eligibility, giving rationale. | Page 7 |
| Information sources | 7 | Describe all information sources (e.g., databases with dates of coverage, contact with study authors to identify additional studies) in the search and date last searched. | Page 7 and Supplementary Table 2 |
| Search | 8 | Present full electronic search strategy for at least one database, including any limits used, such that it could be repeated. | Page 6-7 and Supplementary Table 2 |
| Study selection | 9 | State the process for selecting studies (i.e., screening, eligibility, included in systematic review, and, if applicable, included in the meta-analysis). | Page 7 and Figure 1 |
| Data collection process | 10 | Describe method of data extraction from reports (e.g., piloted forms, independently, in duplicate) and any processes for obtaining and confirming data from investigators. | Page 7 |
| Data items | 11 | List and define all variables for which data were sought (e.g., PICOS, funding sources) and any assumptions and simplifications made. | Page 7 |
| Risk of bias in individual studies | 12 | Describe methods used for assessing risk of bias of individual studies (including specification of whether this was done at the study or outcome level), and how this information is to be used in any data synthesis. | NA |
| Summary measures | 13 | State the principal summary measures (e.g., risk ratio, difference in means). | Page 7 Method Section |
| Synthesis of results | 14 | Describe the methods of handling data and combining results of studies, if done, including measures of consistency (e.g., I^2^) for each meta-analysis. | Page 7 Method Section |

Page 1 of 2

| **Section/topic** | **#** | **Checklist item** | **Reported on page #** |
| --- | --- | --- | --- |
| Risk of bias across studies | 15 | Specify any assessment of risk of bias that may affect the cumulative evidence (e.g., publication bias, selective reporting within studies). | Page 8  Method Section |
| Additional analyses | 16 | Describe methods of additional analyses (e.g., sensitivity or subgroup analyses, meta-regression), if done, indicating which were pre-specified. | Page 8  Method Section |
| **RESULTS** | | |  |
| Study selection | 17 | Give numbers of studies screened, assessed for eligibility, and included in the review, with reasons for exclusions at each stage, ideally with a flow diagram. | Figure 1 |
| Study characteristics | 18 | For each study, present characteristics for which data were extracted (e.g., study size, PICOS, follow-up period) and provide the citations. | Table 1 |
| Risk of bias within studies | 19 | Present data on risk of bias of each study and, if available, any outcome level assessment (see item 12). | NA |
| Results of individual studies | 20 | For all outcomes considered (benefits or harms), present, for each study: (a) simple summary data for each intervention group (b) effect estimates and confidence intervals, ideally with a forest plot. | Figure 2-7 Table 2-6 |
| Synthesis of results | 21 | Present results of each meta-analysis done, including confidence intervals and measures of consistency. | Figure 2-7 Table 2-6 |
| Risk of bias across studies | 22 | Present results of any assessment of risk of bias across studies (see Item 15). | Page 14-25 results are presented in each subheadings |
| Additional analysis | 23 | Give results of additional analyses, if done (e.g., sensitivity or subgroup analyses, meta-regression [see Item 16]). | Page 14 the fourth paragraph |
| **DISCUSSION** | | |  |
| Summary of evidence | 24 | Summarize the main findings including the strength of evidence for each main outcome; consider their relevance to key groups (e.g., healthcare providers, users, and policy makers). | Page 26 |
| Limitations | 25 | Discuss limitations at study and outcome level (e.g., risk of bias), and at review-level (e.g., incomplete retrieval of identified research, reporting bias). | Page 26-27 |
| Conclusions | 26 | Provide a general interpretation of the results in the context of other evidence, and implications for future research. | Page 26-27 |
| **FUNDING** | | |  |
| Funding | 27 | Describe sources of funding for the systematic review and other support (e.g., supply of data); role of funders for the systematic review. | Page 28 |

*From:*  Moher D, Liberati A, Tetzlaff J, Altman DG, The PRISMA Group (2009). Preferred Reporting Items for Systematic Reviews and Meta-Analyses: The PRISMA Statement. PLoS Med 6(7): e1000097. doi:10.1371/journal.pmed1000097

For more information, visit: **www.prisma-statement.org**.

Page 2 of 2

# **Table 2 Full Search Strategy**

| Source | Key Words | Comment |
| --- | --- | --- |
| Web of Science | PrEP related terms (pre-exposure prophylaxis OR preexposure prophylaxis OR antiretroviral prophylaxis OR preexposure chemoprophylaxis OR chemoprevention OR PrEP OR Truvada), disease terms (HIV OR AIDS) and target population terms (MSM OR gay OR men have sex with men). | We conducted twice searching using these key words (May, 10, 2017 and Nov.31,17,2017).Meanwhile e-mail alert was also screened. (Primary Source) |
| Pubmed | PrEP related terms (pre-exposure prophylaxis OR preexposure prophylaxis OR antiretroviral prophylaxis OR preexposure chemoprophylaxis OR chemoprevention OR PrEP OR Truvada), disease terms (HIV OR AIDS) and target population terms (MSM OR gay OR men have sex with men) | We conducted twice searching using these key words (May,10, 2017 and Nov.31,17,2017). Meanwhile e-mail alert was also screened. (Primary Source) |
| Google Scholar | PrEP related terms: PrEP OR preexposure prophylaxis  Disease term: HIV OR AIDS | Google scholar was used as an important supplementary search, so we did not use narrow key words. And we screened related items and related information which were not included in the primary search. (Supplementary Source) |
| Clinicaltrials.gov | PrEP related terms: PrEP OR preexposure prophylaxis  Disease term: HIV OR AIDS | Most clinical studies are registered in Clinicaltrials.gov. And we screened related registration items which wre not included in the primary search.(Supplementary Source) |
| Reference List |  | The reference list of included papers was screened. (Supplementary Source) |
| Review article |  | PrEP-related guidelines (such as CDC,WHO) and PrEP-related review articles were screened. |

# **Table 3 Summary of Grade 3 or 4 adverse event**

| Study | Outcome | Comment |
| --- | --- | --- |
| CDC Safety Study | 62 grade 3 or 4 AEs occurred among 37(9.9%) participants (36 in TDF; 26 in placebo) | Rate Ratio(95% CI)=1.13(0.61,2.11),p=0.703 |
| Ipergay | Any grade 3 or 4 AEs occurred among 34 participants(19 participants in TDF/FTC;15 participants in placebo) | p=0.45 |
| iPrEx | 533 grade 3 or 4 AEs occurred among 315 participants(151 participants in TDF/FTC;164 participants in placebo) | p=0.51 |
| Project PrePare | TDF/FTC: one increased blood bilirubin, one migraine headache and two instances of decreased creatinine clearance in one participant.  Placebo: one psychiatric hospitalization | NR |
| MTN-017 | Six grade 3 or 4 AEs | NR |
| HPTN-069 | 20 participants with 23 grade 3 or 4 AEs | NR |
| ATN110YSMS | 3grade 3 AEs (nausea,weight loss and headache) among 3 participants(related to study); 21 grade 3 or higher AE among 15 participants(unrelated to study) | NR |
